# Aneurysm Location Affects Clinical Course and Mortality in Patients With Subarachnoid Hemorrhage

**DOI:** 10.3389/fneur.2022.846066

**Published:** 2022-03-14

**Authors:** Jennifer Göttsche, Andras Piffko, Tobias F. Pantel, Manfred Westphal, Lasse Dührsen, Patrick Czorlich, Thomas Sauvigny

**Affiliations:** Department of Neurosurgery, University Medical Center Hamburg-Eppendorf, Hamburg, Germany

**Keywords:** subarachnoid hemorrhage, location, cerebral aneurysm, mortality, neurointensive care

## Abstract

**Objective:**

The influence of preexisting factors on the clinical course of patients with subarachnoid hemorrhage (SAH), such as patient age, arterial hypertension, and aneurysm characteristics, is still a matter of debate. However, the specific impact of the exact aneurysm location has not received adequate attention. Therefore, the aim of this study was to investigate the influence of aneurysm location as a preexisting factor on the clinical course and mortality.

**Methods:**

The data of consecutive patients with aneurysmal SAH who were treated from October 2010 to July 2020 were retrospectively analyzed. We distinguished four aneurysm locations: the anterior complex, internal carotid artery (ICA), middle cerebral artery (MCA), and posterior circulation. Logistic regression analysis and receiver operating characteristics were used to investigate the influence of aneurysm location on the occurrence of acute hydrocephalus, Delayed Cerebral Ischemia (DCI), neurological outcome, and in-hospital mortality. Neurological outcome was assessed 3 months after discharge using the Glasgow Outcome Scale.

**Results:**

A total of 603 patients were included in this study. Patients with MCA aneurysms were 2.52 times less likely to develop acute hydrocephalus compared to patients with anterior complex aneurysms (*p* = 0.001). Delayed cerebral ischemia occurred most frequently in patients with an anterior complex aneurysm and least frequently in MCA aneurysms (*p* = 0.014). In ICA aneurysms, mortality was 2.56-fold higher than in patients with aneurysms of the anterior complex (*p* = 0.006). An additional ROC analysis showed a good prediction for in-hospital mortality when taking the aneurysm's location into account [AUC.855 (CI.817 −0.893)].

**Conclusions:**

The aneurysm's location proved to be a significant predictor of acute hydrocephalus, DCI, and in-hospital mortality, demonstrating the impact of this preexisting biological factor on the course of SAH.

## Introduction

Aneurysmal subarachnoid hemorrhage (SAH) remains a disease with high morbidity and mortality rates (1). Recent improvements in therapy have had only minor impact on the outcome of this patient collective and the determinant of the clinical course still needs the exploration of more parameters. Thus, there is a need to evaluate the impact of pre-existing or acquired risk factors.

Many parameters have been described, such as blood clot thickness and scores for the extent of hemorrhage severity [e.g., World Federation of Neurological Surgeons (WFNS) score] (2).

In addition, there is evidence that several other pre-existing factors, independent of the hemorrhage severity, may also have an impact on outcome including patient age, aneurysm size and location, and a history of hypertension (2–5).

As for location, it has been repeatedly postulated that patients with SAH from a posterior circulation aneurysm are likely to have a worse outcome (3, 6, 7). However, apart from this categorical subdivision, a more refined analysis related to the different vascular territories is needed to explore possible prognostic clues (4, 5, 8). Such an independent predictive value of aneurysmal location on the clinical course and mortality has not yet been conclusively demonstrated. Therefore, the aim of our work was to investigate whether in a large institutional cohort within a homogenous treatment regimen, the aneurysm's location, as a biological factor, can provide a clue for estimating clinical course and in-hospital mortality. This knowledge could improve the prognostic estimation complementary to existing scores.

## Materials and Methods

All patients treated at our institution with an aneurysmal SAH from October 2010 to July 2020 who were 18 years or older were included in this retrospective single-center study.

The aneurysmal nature of the hemorrhage was verified by cerebral digital subtraction angiography, cranial CT (cCT) angiography, and/or MRI angiography. Patients in whom no aneurysm could be identified or who died prior to sufficient diagnostics were excluded from further analysis. When multiple aneurysms were identified, the data shown refer to the symptomatic aneurysm.

The demographic data and patients' cardiovascular risk factors were collected as well as aneurysm specific data. Clinical data including scores for severity of SAH (WFNS and Fisher score) were assessed at the first encounter with a physician. Furthermore, data on the clinical course, such as development of an acute hydrocephalus, type of aneurysm treatment (microsurgical vs. endovascular procedures), development of delayed cerebral ischemia (DCI) according to Vergouwen et al. (9) and outcome/mortality, were collected. We perform CT imaging in all SAH patients 24 h after aneurysm securing. Furthermore, multimodal monitoring of the patients is performed by means of transcranial Doppler sonography, which was performed at least once a day, as well as P_bt_O_2_-measurement, if applicable. Vasospasm is suspected by transcranial Doppler sonography (TCD) if the mean flow velocity in the middle cerebral artery is above 140 cm/s and above 120 cm/s in the anterior cerebral artery, respectively. In case of TCD suspicion of vasospasm, we perform a CT scan including CT angiography and CT perfusion imaging according to our local treatment protocol. In addition, the MAP is increased to values of 90–100 mmHg and nimodipine is administered continuously intravenously to ensure the dosage (10). If a P_bt_O_2_-measurement is performed and a drop of values below 20 mmHg was detected, multimodal imaging as described before was carried out, followed by the above-mentioned therapeutic interventions.

The outcome data were extracted from the electronic patient file which contained the examination results of either visits at our outpatient clinic and/or detailed reports from rehabilitation units. Acute hydrocephalus was diagnosed by experienced senior physicians on the base of image morphology in correlation with the clinical presentation of patients.

Regarding the location of aneurysms, four groups were distinguished in analogy to the classification in the Subarachnoid Hemorrhage International Trialists (SAHIT) study: anterior complex (including the anterior cerebral artery, anterior communicating artery (AcoA), and pericallosal artery), internal carotid artery [ICA, including posterior communicating artery (PcoA)], middle cerebral artery (MCA), and posterior circulation (including basilar artery, vertebral artery, posterior inferior cerebellar artery (PICA), superior cerebellar artery (SUCA), anterior inferior cerebellar artery (AICA), and posterior cerebral artery) (4, 5).

Statistical analyses were performed using the IBM SPSS^®^ version 25 (IBM Corporation, Armonk, NY, USA). The statistical level of significance was set to *p* < 0.05. The statistical analysis of the data was performed by univariable analysis using the Fisher's exact test, chi-square test, Kruskal-Wallis test, or ANOVA tests depending on the scale and distribution of measurements. In a next step, acute hydrocephalus, DCI, favorable outcome, and in-hospital mortality were pre-defined as outcome parameters of interest. For each outcome parameter, a logistic regression analysis was performed to determine the independent predictive value of aneurysm location and covariates at baseline which have been described in the literature and showed a significant association in univariate analysis. All shown odds ratios (OR) are adjusted ORs for covariates calculated in the respective regression model.

A favorable outcome was defined as a value greater than or equal to 4 on the Glasgow Outcome Scale (GOS) 3 months after discharge.

In order to evaluate the predictive performance of known predictors combined with aneurysm location, receiver operating characteristic (ROC) curves and corresponding areas under the curve (AUC) were calculated.

This study was conducted according to the Declaration of Helsinki, local and institutional laws, and was reported to the local ethical committee (No. WF-065/21).

## Results

Between October 2010 and July 2020, 633 patients with aneurysmal SAH were treated in our institution. A total of 603 patients were eligible for further analyses.

The mean age of patients was 55.1 ± 13.5 years. A total of 40.1% (*n* = 242) of the aneurysms were located in the anterior complex and in 93 cases, the aneurysm was located in the posterior circulation. The mean diameter of aneurysms was 7.2 mm (± 5.2). Details are presented in Table 1.

**Table 1:** Demographic, clinical and aneurysm characteristics.

**Sex–no. (%)**	
** Male**	**202 (33.5)**
** Female**	**401 (66.5)**
**Age (years)–mean (sd)**	**55.1 (13.5)**
**Aneurysm location–no. (%)**	
** Anterior complex**	**242 (40.1)**
** ACA**	**17**
** Pericallosal artery**	**20**
** AcoA**	**205**
** ICA**	**144 (23.9)**
** PcoA**	**75**
** MCA**	**124 (20.6)**
** Posterior circulation**	**93 (15.4)**
** Basilar artery**	**57**
** Vertebral artery**	**14**
** PICA**	**17**
** SUCA**	**4**
** AICA**	**1**
**Aneurysm diameter (mm)–mean (sd)**	**7.2 (5.2)**
**Anterior complex**	**5.6 (3.4)**
**ICA**	**8.6 (6.4)**
**MCA**	**7.9 (5.2)**
**Posterior circulation**	**8.2 (6.2)**
**WFNS *grade–*no. (%)**	
**1**	**285 (47.3)**
**2**	**50 (8.3)**
**3**	**37 (6.1)**
**4**	**81 (13.4)**
**5**	**145 (24.0)**
**Missing**	**5 (0.8)**
**Initial pupil status–no. (%)**	
**Normal**	**504 (83.6)**
**Anisocoria**	**49 (8.1)**
**Bilaterally dilated**	**19 (3.2)**
**Missing**	**31 (5.1)**
**CVRF–no. (%)**	
**Nicotine abuse**	**142 (23.5)**
**Arterial hypertension**	**247 (41.0)**
**Acute hydrocephalus–no. (%)**	
**EVD/LD**	**393 (65.2)**
**Shunt-dependent chronic**	
**Hydrocephalus–no. (%)**	**75 (12.4)**
**Treatment–no. (%)**	
** endovascular**	**400 (66.3)**
** –Coilembolization**	**344 (57.0)**
** –WEB-Device**	**44 (7.3)**
** –Flow diverter**	**12 (2.0)**
**Microsurgical clipping**	**171 (28.4)**
**Not eligible for treatment**	**31 (5.1)**
**DCI–no. (%)**	**208 (34.5)**
**Stay on ICU (days)–mean (sd)**	**18.4 (11.4)**
**Deceased during hospital stay–no. (%)**	**117 (19.4)**
**GOS after 3 months**	
**1**	**129 (21.4)**
**2**	**48 (8.0)**
**3**	**87 (14.4)**
**4**	**114 (18.9)**
**5**	**223 (37.0)**
**Lost to-follow-up**	**2 (0.3)**

*ACA, anterior cerebral artery; AcoA, anterior communicating artery; AICA, anterior inferior cerebellar artery; CVRF, cardiovascular risk factors; EVD, external ventricular drain; GOS, Glasgow outcome scale; ICA, internal carotid artery; LD, lumbar drain; MCA, middle cerebral artery; PcoA, posterior communicating artery; PICA, posterior inferior cerebellar artery; SUCA, superior cerebellar artery; VP-Shunt, ventriculoperitoneal Shunt; WFNS, World Federation of Neurosurgical Societies*.

A total of 117 (19.4%) patients died during their hospital stay with the majority of patients (94.9%) dying from SAH-specific causes (evidence of irreversible loss of brain function; active discontinuation of life-support-based imaging results and presumed will of the patient). In six cases, there were non-SAH specific causes (sepsis with multiorgan failure in five cases and circulatory failure due to complete intestinal ischemia in one case). Of these non-SAH specific deaths, three cases were in patients with aneurysms of the anterior complex, one patient with aneurysm of the ICA, and two patients with the aneurysm of the MCA.

Aneurysms in the anterior complex represented the most frequent location in both sexes but were significantly more frequent in male than in female patients. Posterior circulation aneurysms were predominantly treated by endovascular means (95.2% of all treated aneurysms in this location) as were aneurysms of the ICA (84.3%) and aneurysms of the anterior complex (79.4%). MCA aneurysms, on the other hand, were treated mainly by microsurgical clipping (80.5%). Depending on aneurysm location, significant differences in the clinical course were found on univariable analyses (Table 2). Patients with an aneurysm of the posterior circulation as the cause of hemorrhage had the highest rate of acute hydrocephalus (79.6%, *p* < 0.001). Delayed cerebral ischemia occurred most frequently in patients with an anterior complex aneurysm (38.4%, *p* = 0.041). Vertebrobasilar aneurysms showed the highest in-hospital mortality at 26.9%, followed by ICA aneurysms (26.4%). Aneurysm of the anterior complex and MCA aneurysms showed the lowest mortality (15.3 and 13.7%; *p* = 0.005). However, WFNS grade did not differ significantly between locations (*p* = 0.622). Next, regression analyses were used to determine the independent predictive value of aneurysm location and predefined parameters described in the literature regarding mortality and functional outcome in SAH patients (Table 3) (11–19). Preexisting characteristics or clinical findings quantifying the bleeding itself (WFNS grade, Fisher grade, and pupil status) were not considered as dependent (target) variables but were included as covariates. Additionally, all parameters at baseline listed in Table 1 were tested for an association with DCI or acute hydrocephalus using univariate analysis. Here, WFNS (*p* < 0.001), Fisher grade (*p* < 0.001), and aneurysm location (*p* < 0.001) showed a significant association with acute hydrocephalus and were, therefore, included in the regression analysis (see Table 3). Age, sex, arterial hypertension, initial pupil status, and aneurysm diameter did not reach statistical significance.

**Table 2:** Univariable analysis of clinical parameters dependent on aneurysm location.

	**Aneurysm location**				***p*-value**
	**Anterior ICA**	**MCA**	**Posterior circulation**	**Complex**	
Sex–no. (%)					
Male	100 (41.3)	33 (22.9)	36 (29.0)	33 (35.5)	0.002
Female	142 (58.7)	111(77.1)	88 (71.0)	60 (64.5)	
Arterial hypertension–no. (%)	96 (39.7)	54 (37.5)	51 (41.1)	46 (49.5)	0.286
WFNS *grade–*no. (%)					0.622
1	125 (51.7)	60 (41.7)	62 (50.0)	38 (40.9)	
2	19 (7.9)	14 (9.7)	7 (5.6)	10 (10.8)	
3	11 (4.5)	11 (7.6)	10 (8.1)	5 (5.4)	
4	32 (13.2)	20 (13.9)	17 (13.7)	12 (12.9)	
5	52 (21.5)	39 (27.1)	27 (21.8)	27 (29.0)	
Missing	3 (1.2)		1 (0.8)	1 (1.1)	
Initial pupil status–no. (%)					
Normal	211 (87.2)	114 (79.2)	104 (83.9)	75 (80.6)	0.023
Anisocoria	13 (5.4)	20 (13.9)	7 (5.6)	9 (9.7)	
Bilaterally dilated	8 (3.3)	1 (0.7)	5 (4.0)	5 (5.4)	
Missing	10 (4.1)	9 (6.3)	8 (6.5)	4 (4.3)	
Rebleed during hospital stay	38 (15.7)	22 (15.3)	12 (9.7)	12 (12.9)	0.366
Fisher					0.024
1	8 (3.5)	8 (6.1)	4 (3.6)	4 (4.8)	
2	28 (12.3)	14 (10.6)	16 (14.3)	2 (2.4)	
3	48 (21.1)	19 (14.4)	25 (22.3)	11 (13.1)	
4	144 (63.2)	91 (68.9)	67 (59.8)	67 (79.8)	
Missing	14 (6.1)	12 (9.1)	12 (10.7)	9 (10.7)	
Acute hydrocephalus–no. (%)	158 (65.3)	101 (70.1)	60 (48.4)	74 (79.6)	<0.001
Shunt-dependent chronic hydrocephalus–no. (%)	33 (13.6)	16 (11.1)	11 (8.9)	15 (16.1)	0.334
Treatment–no. (%)					<0.001
Coiling	158 (65.3)	99 (68.8)	20 (16.1)	67 (72.0)	
Flow diverter	0 (0.0)	4 (2.8)	0 (0.0)	8 (8.6)	
WEB device	27 (11.2)	10 (6.9)	3 (2.4)	4 (4.3)	
Microsurgical clipping	48 (19.8)	24 (16.7)	95 (76.6)	4 (4.3)	
None	9 (3.7)	7 (4.9)	6(4.8)	10 (10.8)	
DCI–no. (%)	93 (38.4)	52 (36.1)	31 (25.0)	32 (34.4)	0.041
Deceased during hospital stay–no. (%)	37 (15.3)	38 (26.4)	17 (13.7)	25 (26.9)	0.005
GOS at discharge–no. (%)					0.198
1	37 (15.3)	38 (26.4)	17 (13.7)	25 (26.9)	
2	21 (8.7)	9 (6.3)	12 (9.7)	6 (6.5)	
3	55 (22.7)	24 (16.7)	24 (19.4)	14 (15.1)	
4	57 (23.6)	29 (20.1)	31 (5.1)	22 (23.7)	
5	72 (29.8)	44 (30.6)	40 (6.6)	26 (28.0)	
GOS after 3 months–no. (%)					0.368
1	43 (17.8)	41 (28.5)	19 (15.4)	26 (28.0)	
2	21 (8.7)	11 (7.6)	9 (7.3)	7 (7.5)	
3	39 (16.2)	19 (13.2)	17 (13.8)	12 (12.9)	
4	46 (19.1)	22 (15.3)	29 (23.6)	17 (18.3)	
5	92 (38.2)	51 (35.4)	49 (39.8)	31 (33.3)	
Lost to follow-up	1 (0.4)		1 (0.8)		

*%, % within aneurysm location; DCI, delayed cerebral ischemia; IQR, interquartile range; sd, standard deviation*.

**Table 3:** Logistic regression analyses.

**Tested variables**	**OR**	**CI**	***p*-value**
Mortality			
Age	1.027	1.007–1.047	0.008
WFNS	1.655	1.395–1.964	<0.001
Pupil Status normal (ref)			<0.001
Anisocoria	3.318	1.589–6.927	0.001
Bilaterally dilated	20.291	5.193–79.285	<0.001
Rebleed	3.925	2.054–7.498	<0.001
Location anterior complex (ref)			0.005
MCA	1.063	0.478–2.362	0.881
ICA	2.556	1.316–4.966	0.006
Posterior circulation	2.923	1.368–6.247	0.006
Acute hydrocephalus			
WFNS	1.404	1.229–1.603	<0.001
Fisher	2.289	1.796–2.918	<0.001
Location anterior complex (ref)			<0.001
MCA	0.397	0.235–0.673	0.001
ICA	1.203	0.704–2.055	0.498
Posterior circulation	1.637	0.841–3.188	0.147
DCI			
WFNS	1.098	0.984–1.226	0.094
Fisher	1.056	0.821–1.359	0.671
Acute hydrocephalus	2.273	1.466–3.522	<0.001
location anterior complex (ref)			0.100
MCA	0.518	0.306–0.875	0.014
ICA	0.774	0.487–1.231	0.279
Posterior circulation	0.745	0.436–1.274	0.282
Favorable outcome after 3 months			
Age	0.959	0.944–0.975	<0.001
WFNS	0.606	0.531–0.693	<0.001
Fisher	0.748	0.569–0.983	0.038
DCI	0.271	0.175–0.420	<0.001
Location anterior complex (ref)			0.510
MCA	0.973	0.535–1.769	0.929
ICA	0.671	0.387–1.164	0.156
Posterior circulation	0.960	0.503–1.832	0.902

For DCI as a dependent variable, WFNS grade (*p* = 0.008), Fisher grade (*p* = 0.005), acute hydrocephalus (*p* < 0.001), and aneurysm location (*p* = 0.041) revealed a significant association in univariate testing while age, sex, arterial hypertension, initial pupil status, treatment modality, occurrence of rebleeding, and aneurysm diameter did not reach significance.

Regression analysis revealed WFNS grade, patient age, pupil status, occurrence of rebleed, and aneurysm location each as an independent predictor of in-hospital mortality.

Patients with aneurysms of the posterior circulation were 2.92-fold more likely to die than patients with aneurysms of the anterior complex (*p* = 0.006; OR: 2.92; CI: 1.37–6.25). Patients with ICA aneurysms were 2.56-fold more likely to die (*p* = 0.006; OR: 2.56; CI: 1.32–4.97).

In addition to mortality, further clinical events were investigated. Fisher grade and WFNS grade as well as aneurysm location were independent predictors of acute hydrocephalus in the logistic regression analysis (Table 3). With respect to the reference location (anterior complex), patients with an aneurysm of the MCA were 2.52 (*p* = 0.001; OR: 0.39; CI: 24–0.67) times less likely to develop acute hydrocephalus.

In the regression analysis, acute hydrocephalus was found to be an independent predictor of DCI. However, SAH patients with an MCA aneurysm were 1.93 times less likely to present with DCI during the course than patients with an aneurysm of the anterior complex (*p* = 0.014; OR: 0.52; CI: 31–0.86).

A total of 337 patients had a favorable outcome (GOS 4+5) after 3 months. Logistic regression showed age, WFNS and Fisher grade, and absence of DCI as predictors of a favorable outcome at 3 months. The location of the aneurysm had no independent predictive value.

### Receiver Operating Characteristics

**Figure 1:**
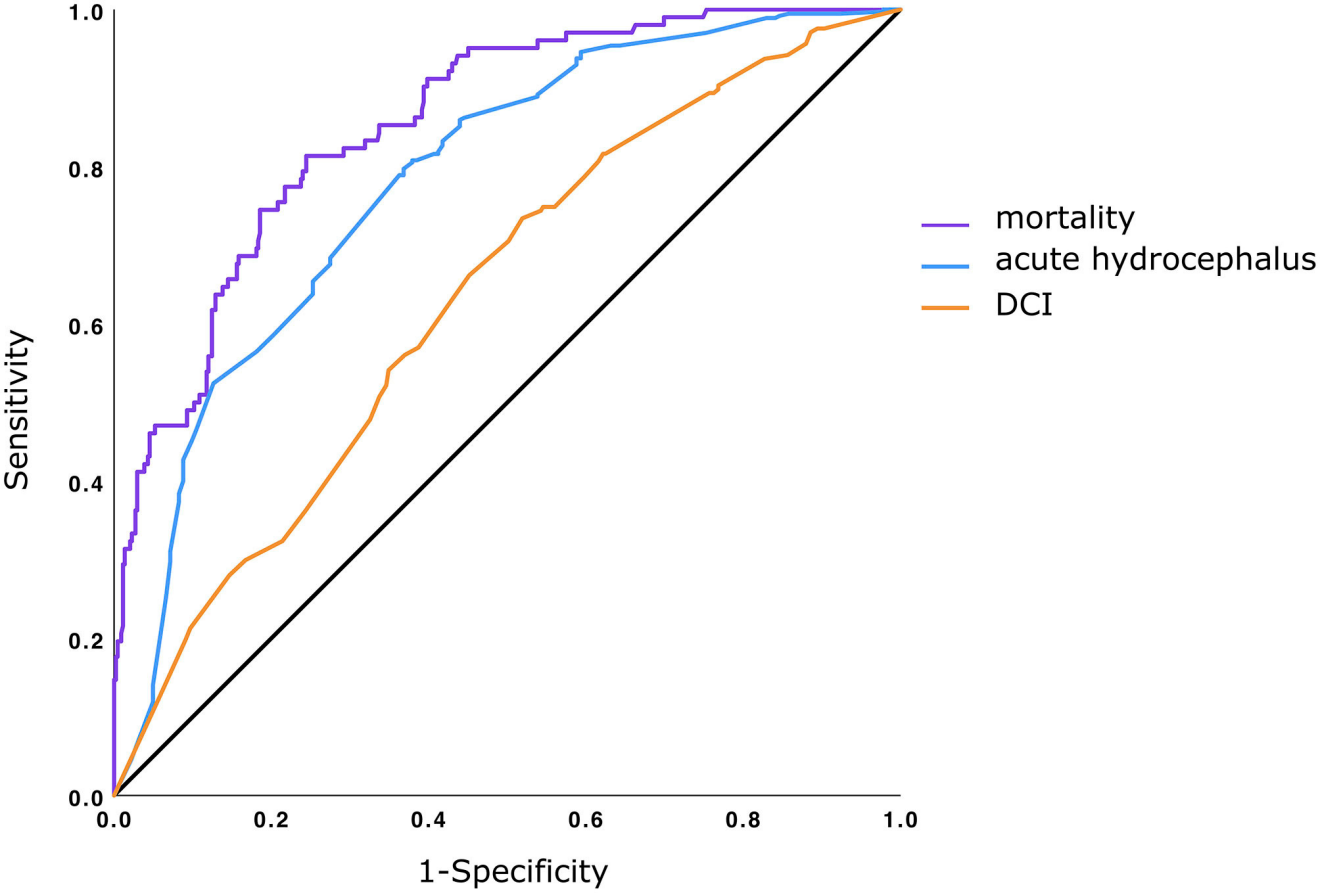
Receiver operating characteristics (ROC) for prediction of mortality, acute hydrocephalus, and delayed cerebral ischemia (DCI). Purple: ROC predicting in-hospital-mortality (based on regression analysis using age, World Federation of Neurological Surgeons (WFNS) grade, pupil status, rebleed, and aneurysm location as covariates). AUC 0.855; CI 0.817–0.893. Blue: ROC predicting an acute hydrocephalus (age, sex, arterial hypertension, WFNS and Fisher grade, and aneurysm location as covariates). AUC 0.782; CI 0.740–0.824. Orange: ROC predicting DCI (based on age, sex, WFNS and Fisher grade, arterial hypertension, and aneurysm location as covariates). AUC 0.634; CI 0.587–0.681.

Receiver operating curves were calculated for all outcome variables for which location was predictive (in-hospital mortality, acute hydrocephalus, and DCI) using the parameters of their respective regression analysis. For the prediction of in-hospital mortality, AUC was 0.86 (CI.82–0.89) (shown in Figure 1). The AUC for acute hydrocephalus was 0.78 (CI.74–0.82) and for the occurrence of DCI was 0.63 (CI.58–0.68).

## Discussion

Scores, such as WFNS grading, represent the gold standard in prognostic assessment in SAH patients (20, 21). In recent years, new scores have added more parameters to increase the validity including pupil status and occurrence of rebleed (14, 15).

The relevance of the exact location of the symptomatic aneurysm as a preexisting biological condition in relation to the prognosis has not received adequate attention (2, 22).

Some studies were able to show that SAH patients with an aneurysm of the posterior circulation have a worse functional outcome than patients with an anterior circulation aneurysm (3, 8, 23). However, a precise breakdown of aneurysm location is often missing (14, 24). The effect of aneurysmal location on other prognostically relevant factors such as the occurrence of DCI is even less known (11, 25).

The aim of this study was to evaluate whether the distinct location of an aneurysm in SAH patients has an influence on the clinical course and in-hospital mortality.

Konczalla et al. (12) found an association between aneurysm location at carotid bifurcation and the incidence of developing early hydrocephalus as a sign of early brain injury. In our cohort, the risk of developing acute hydrocephalus was highest in patients with the rupture of a posterior circulation aneurysm. A possible explanation is the high proportion of Fisher grade IV hemorrhages in cases with posterior circulation aneurysms.

WFNS and Fisher grade have also been associated with the development of acute hydrocephalus which could be reproduced in our cohort (13, 26).

Similarly, previously described parameters predicting the mortality and outcome in SAH patients were examined such as age, WFNS score, pupil status, and rebleed (14–16). All these factors reported in the literature, as well as aneurysm location, were also shown to be predictive in our analysis. The location of the aneurysm is known to be related to the risk of rupture (17). Increased mortality in the event of rupture in ICA aneurysms and posterior circulation aneurysms may represent another component in decision-making regarding the treatment of unruptured aneurysms. A treatment recommendation score already includes the factor “reduced quality of life” due to a fear of rupture (27). Our data support location as an independent parameter to estimate the consequences of a possible hemorrhage at a time where the severity of bleeding cannot be foreseen.

Lee et al. (11) were able to show that the rupture of an anterior circulation aneurysm was associated with a higher risk for DCI.This finding was reproduced in our cohort, as patients with an anterior complex aneurysm had nearly twice the risk of developing DCI compared with patients with an MCA aneurysm. Our data show essentially comparable DCI risks for patients with aneurysms of the anterior complex, ICA, and posterior circulation. Only MCA aneurysms had a significantly lower DCI rate. One possible explanation could be the high rate of surgically treated MCA aneurysms (80.5%) in our cohort because it is common practice at our institution to administer local vasodilators during clipping, which could reduce the DCI rate (28).

Receiver operating curves showed only a small AUC for DCI. Efforts have been described in the literature to predict DCI by means of a clinical score (11). Very low scores correctly predicted a low probability of DCI, but the overall positive predictive value for the presence of DCI was moderate. The multifactorial nature of DCI seems to make the prediction of this event more difficult than for other clinical endpoints. Although in patients with aneurysms of the anterior complex, DCI occurred more frequently. These patients showed a lower in-hospital mortality rate which is consistent with a low association between the occurrence of DCI and in-hospital mortality reported in the literature (16, 24). However, as described in the literature, we also identified DCI as a predictor of unfavorable outcome at 3 months in logistic regression. Aneurysm location did not influence functional outcome, but age, WFNS grade, and Fisher grade were essential predictors in our study as well.

This finding suggests that the neurological outcome depends, to a certain extent, on the course after hemorrhage as well as on initial non-modifiable parameters such as bleeding severity. In contrast, mortality, which in many cases occurs during the early phase after hemorrhage (death was ascertained after a median of 8 days), seems to depend largely on preexisting factors including aneurysm location.

The retrospective analysis of the database and the single-center design of this study present limitations. Another limitation is the relatively long period of time covered by the data, which means that although a larger cohort was studied, performance bias cannot be ruled out because treatment algorithms have changed over the years. For example, there is no longer any hemodilution or hypervolemia, which in some cases was still the case at the beginning of the study, since efficacy of triple-H could not be verified (29, 30). Furthermore, the detection of vasospasm may have improved through the increasing use of brain tissue oxygenation probes in recent years.

In addition, the location has a clear influence on the type of treatment (as shown in Table 2); the influence of the treatment modality is difficult to estimate. In order to be able to narrow this down further, the treatment modality would also have to be examined in the respective subgroups.

Furthermore, it remains to be noted that neurological and clinical complications not studied in this work, such as intracranial hypertension, seizures, ventriculitis, and pulmonary complications, also correlate with mortality and clinical outcome in SAH patients. These factors represent a potential bias.

In summary, we were able to show significant differences in the clinical course and in-hospital mortality rates with respect to aneurysm location. Aneurysms of the internal carotid artery and posterior circulation aneurysms were associated with the highest case fatality rates. The risk of occurrence of an acute hydrocephalus was highest for aneurysms of the posterior circulation. Aneurysms of the MCA showed the lowest risk of developing DCI.

The rationale to treat asymptomatic aneurysms is mainly based on two considerations: the risk of rupture and the associated risk of serious neurological damage. Therefore, our data may have an impact on the second aspect since aneurysm location is already determined at the time of diagnosis of the unruptured aneurysms. This demonstrates the independent relevance of underlying biological factors to estimate the prognosis and to advise patients more precisely on the consequences of a possible bleeding.

## Data Availability Statement

The raw data supporting the conclusions of this article will be made available by the authors, without undue reservation.

## Ethics Statement

The studies involving human participants were reviewed and approved by Local Ethics Committee Ärztekammer Hamburg Weidestr. 122b 22083 Hamburg. Written informed consent for participation was not required for this study in accordance with the national legislation and the institutional requirements.

## Author Contributions

JG supervised the study, designed the study question, interpreted the data, and drafted the manuscript. AP and TP collected the data. MW contributed to the data interpretation. LD and PC helped to interpret the data and conceive the study questions. TS contributed to the overall design of the study, supervised the study, conceived the study question, designed the analysis plan, and analyzed and interpreted the data. All authors revised the manuscript.

## Conflict of Interest

The authors declare that the research was conducted in the absence of any commercial or financial relationships that could be construed as a potential conflict of interest.

## Publisher's Note

All claims expressed in this article are solely those of the authors and do not necessarily represent those of their affiliated organizations, or those of the publisher, the editors and the reviewers. Any product that may be evaluated in this article, or claim that may be made by its manufacturer, is not guaranteed or endorsed by the publisher.
